# Information Preference and Information Supply Efficiency Evaluation before, during, and after an Earthquake: Evidence from Songyuan, China

**DOI:** 10.3390/ijerph182413070

**Published:** 2021-12-11

**Authors:** Shasha Li, Xinyu Peng, Ruiqiu Pang, Li Li, Zixuan Song, Hongying Ye

**Affiliations:** 1 Jangho Architecture College, Northeastern University, Shenyang 110169, China; lishasha@mail.neu.edu.cn (S.L.); 20175857@stu.neu.edu.cn (X.P.); lili1118@mail.neu.edu.cn (L.L.); 2 Rosedale Academy Shenyang Campus, Shenyang 110011, China; songzixuan0730@163.com; 3 China Building Technique Group Co., Ltd., Beijing 100013, China; yhycmc@126.com

**Keywords:** earthquake disaster, information preference, information deviation index, crisis stage analysis theory, risk communication

## Abstract

Efficient risk communication is aimed at improving the supply of risk information to meet the information needs of individuals, thus reducing their vulnerability when facing the risk of emergency. There is little information available in the literature regarding information preference from an individual’s need perspective, and there is a lack of differentiation in evaluation between information need and supply. Under the guidance of the crisis stage analysis theory, using multiple response analysis and weighted analysis methods, this study explores earthquake disaster information content and communication channel preferences, and develops an information deviation index (IDI) to evaluate the efficiency of risk communication before, during, and after an earthquake. A questionnaire-based survey of 918 valid respondents in Songyuan, China, which had been hit by a small earthquake swarm, was conducted to provide practical evidence for this study. The results indicated the following. Firstly, the information needs of individuals are highly differentiated in the different stages of an earthquake. From pre-disaster to post-disaster, individuals show a shift in information need from “preparedness and response knowledge” to “disaster information”, then to “disaster information and disaster relief information” in parallel, to “reconstruction and reflection information”. Based on the above analysis, a composition of the main earthquake disaster information is proposed for different stages. Secondly, by measuring the values of the IDI, we found that most individuals’ information needs were met for the earthquake. Thirdly, the TV and the internet were the two preferred commutation channels for acquiring disaster information from among all the effective channels in all the stages.

## 1. Introduction

What is a disaster? “A serious disruption of the functioning of a community or a society at any scale due to hazardous events interacting with conditions of exposure, vulnerability and capacity, leading to one or more of the following: human, material, economic and environmental losses and impacts” [1]. Earthquakes are one of the most difficult hazards to be predicted and can result in massive casualties, property losses, and loss of municipal infrastructure. It is important for individuals to acquire sufficient earthquake disaster information so that, when faced with an earthquake, they are able to make the right decisions and take rapid actions with regard to mitigation measures and adjustments [2,3,4,5,6,7,8,9,10,11,12,13]. Research on the public’s disaster information acquisition content and channel preferences may help to improve the efficiency of information supply. For example, if a communication channel is found to be effective in motivating the public to respond proactively to risk information, but is used sparingly, its effectiveness is limited [14,15]. Alternatively, if the public is eager to learn about some aspect of the risk, but the information is not available when it is released, the communication is also a failure. However, research on public disaster information preferences is often underappreciated [11,12,13].

Information plays an important role in disaster management and is an indispensable resource in disasters [15]. The theme of the World Disasters Report 2005 [16] is “Information in Disasters”, and it systematically reviews the functions, sources, and channels of emergency information. Previous studies on earthquake disaster information mainly focused on two areas. The first area is research on earthquake disaster information classification. Information classification is distinguishing and classifying information in line with certain principles and methods according to the attributes or characteristics of the information content, establishing a certain classification system and arrangement order [17]. Chinese scholars have conducted many studies in the field of earthquake emergency information classification [18,19,20,21,22,23,24]. For instance, Nie et al. [18] proposed a classification system for basic earthquake emergency data. Su et al. [19] divided earthquake emergency information into seventeen categories. Bai et al. [20] classified earthquake emergency site information into four categories: earthquake information, disaster information, emergency response information, and disposal benefit information. Based on the historical earthquake disaster experience, emergency rescue experience and relevant national standards, on-site disaster information was divided into nine categories by Zhu et al. [21]. Dong et al. [22] categorized earthquake emergency disaster information into four categories, namely seismic information, earthquake zone background information, disaster information, and emergency response. Zhang et al. [23] ordered earthquake emergency disaster information into six categories. Su et al. [24] divided earthquake emergency information into eight categories. In addition, Sapountzaki [25] and Fokaefs [26] proposed that earthquake emergency information be grouped into four types of messages. See Table 1 for details.

The classification of earthquake emergency disaster information provides an important reference for our research. However, to summarize the above literature, we found that:

(a) The earthquake disaster information mentioned above only referred to earthquake emergency information after earthquake disasters, and there was a lack of attention paid to information prior to the earthquake. Risk communication spans every phase of the (disaster) risk management cycle, not only the emergency [26]; 

(b) All the earthquake disaster information was collected and extracted from the internet, and such indirectly acquired information cannot reveal the information needs of general public in detail;

(c) It was unclear who the users of the earthquake disaster information were. The users may have come from the government category (i.e., government decision-making departments or decision makers), the industry category (i.e., industry information departments or industry information practitioners, or scientific research and engineering technicians), or the public category (i.e., social public groups or individuals, or mass media) [27]. There is a lack of clarification on the information audience.

The second area is research on the earthquake disaster information needs of a certain group. Jeffrey [28] explored the preferences of information needs in three phases: before, during, and after a crisis, while Rego [29] studied the information needs of disaster managers before and after disasters. Yang and Liu [30] investigated the basic orientation and characteristics of the information needs of the public in a crisis situation, taking the 12 May earthquake in 2008 as an example, and concluded that the public’s information needs were differentiated at different stages of the earthquake. Sha and Xu [31] constructed an information analysis framework for emergency information needs based on the activity theory. Lei et al. [32] proposed a general and systematic framework for emergency information needs. Wang et al. [33] preliminarily summarized the information needs of earthquake emergency rescue, and put forward some suggestions on the information acquisition of major earthquakes and catastrophes. Zhou et al. [34], taking the Ms5.1 Sichuan Qingbaijiang earthquake as an example, studied the information product’ needs of earthquake emergency personnel and scientific research personnel. Zhang et al. [35], taking the members of the earthquake relief headquarters at all levels in Yunnan as the research objects, analyzed their earthquake emergency information needs. Most of the above studies used direct investigation methods to obtain data and to accurately acquire earthquake disaster information needs for a specific population; however, an evaluation of the difference between information need and supply was lacking.

Numerous sources of information are involved in crisis communications, providing information to both at-risk and general audiences [36,37]. However, selecting the most effective channels can still be challenging. Traditional mass media mainly refers to television, radio and newspapers and magazines. Mass media has been found to be the most utilized channel of information dissemination during disasters [12,14,26,38,39,40,41,42,43]. Among them, radio is considered to be the most frequently used information dissemination channel in crisis events [12,14,44,45,46]. Furthermore, of course, there are studies which show that television remains the most common channel that audiences use in times of crisis [26,47,48,49]. Compared with traditional media, social media is also considered to be an important channel [50,51,52,53,54,55,56] for risk information due to its interactivity and speed. In addition to traditional media and new media, another information dissemination channel that cannot be ignored is interpersonal communication, which mainly refers to communication and dissemination among family members, friends, and neighbors. This informal information channel is considered to be one of the most important information sources during disasters [14,57,58]. Moreover, short message service (SMS) is applied widely by agencies worldwide [59], for instance, the US federal emergency management [60,61], Swiss National Weather Agency [62], and the Meteorological Service of New Zealand [61]. Most studies have focused on the information acquisition channels at one stage of a sudden crisis event [14], and discussion of the integrity of information disseminated channels has been missing.

To address these issues, this paper attempts to discuss the following issues: 

(a) From the perspective of the needs of residents in earthquake-stricken areas, we attempt to explore their earthquake disaster information preferences, that is, what information content is needed and how this information should be disseminated. Finally, we propose the ideal composition of main disaster information at different stages of an earthquake;

(b) Risk communication efficiency based on whether the residents’ information needs are being met. We develop an information deviation index (IDI) to evaluate the difference between the information needs and supply. 

This study is presented in six sections. Following the introduction, Section 2 introduces the crisis stage analysis theory through a theoretical lens. Section 3 introduces the study area, the use of the questionnaire-based survey, the research indicators and the data analysis techniques employed. The research findings are highlighted and discussed in Section 4 and Section 5. The Section 6 presents our conclusion, and discusses the limitations, future studies, and the implications of this study.

## 2. Theoretical Lens

The crisis stage analysis theory by Fink [63] is one of the most influential theories in the risk communication field. Fink (2002) proposed a four-stage pattern for crisis communication events. The four stages are crisis prodromal stage, crisis acute stage, crisis chronic stage, and crisis resolution stage. The fundamental purpose of this framework is, according to the basic characteristics of crisis events with stages, to study the characteristics of information communication in crisis events and to acquire coping strategies for crisis communication at different stages. As Fink (2002, p. 21) explained, “By dissecting a crisis and taking a closer, microscopic look at each phase, you may be better able to spot these stages in the future”.

### 2.1. Crisis Prodromal Stage

The crisis prodromal stage is the easiest period in which to resolve crisis events, but it is easily ignored as obvious signs of the event have not yet occurred. In this period, in order to play a preventive and proactive role for the future, the most important work is to prepare for the crisis (which includes psychological and material preparation). Some scholars define the crisis prodromal stage as the period from the occurrence of a disaster to the beginning of the dissemination of disaster information [64].

In this study, the crisis prodromal stage is defined differently and has a longer time front, which refers to the time in daily life before an earthquake occurs; that is, the time before the earthquake disaster occurs in the area where residents live.

### 2.2. Crisis Acute Stage

The sudden outbreak of a crisis event, although short-lived, has a great social impact and causes loss of life and property, with information about the state of affairs spreading rapidly and immediately attracting widespread social attention. It can be said that the crisis outbreak period is the most difficult and urgent period in crisis management [65].

The emergent period in this study refers to the time immediately after the occurrence of an earthquake (i.e., the moment or day of the earthquake), in a state of emergency [66], which is the period from the beginning of the dissemination of earthquake disaster information to the rapid dissemination of earthquake disaster information [64].

### 2.3. Crisis Chronic Stage

When a crisis event enters the chronic stage from the acute stage, the damage caused has already been revealed and the large-scale organization of rescue work has begun. At this time, the public is in a negative mood, and the demand for information is at its maximum. This stage is the most active period for social opinion, so if information from formal channels is not released in a timely manner, all kinds of rumors and gossip may spread and cause group panic.

The chronic stage in this study refers to the earthquake relief period, which emphasizes the stage after the public learns the basic information about the earthquake, including both the post-earthquake emergency period and the relief period after the emergency period [66]. In other words, the stage when earthquake information spreads rapidly until it subsides [64].

### 2.4. Crisis Resolution Stage

In the crisis resolution stage, public attention shifts from the crisis itself to eliminating the effects of the event. In this stage, the crisis is fully controlled and people begin to return to the normal order of life and production, and to rebuild their homes according to the government’s plan.

The crisis resolution stage in this paper refers to the post-disaster reconstruction period, including both the later period of earthquake relief and the reconstruction period after earthquake relief [66]. At this time, the disaster is properly resolved, people’s lives return to normal, material production is restored, social panic is quelled, and the whole society returns to the state before the disaster.

The advantage of Fink’s crisis stage analysis theory is that it provides a broader perspective to understanding the characteristics of the crisis, and it also provides a comprehensive and cyclical approach to the whole process of crisis management. The four stages of a crisis event are periodical processes that show different characteristics [65,67,68,69] (Table 2). Although the proposed stages lack detail and seem to be linearly deterministic, the framework still provides a complete examination of the crisis process, i.e., from its prodrome, development, and sudden change, to its resolution. This is of some relevance to the study of earthquake disaster. In this study, we suggest a timeline for the Songyuan earthquake according to Fink’s crisis stage analysis theory. Further, we discuss the earthquake disaster information content and communication channel preferences, and evaluate the information supply efficiency for the periods when the public perceive different stages of the earthquake disaster.

## 3. Materials and Methods

### 3.1. Study Area

**Figure 1:**
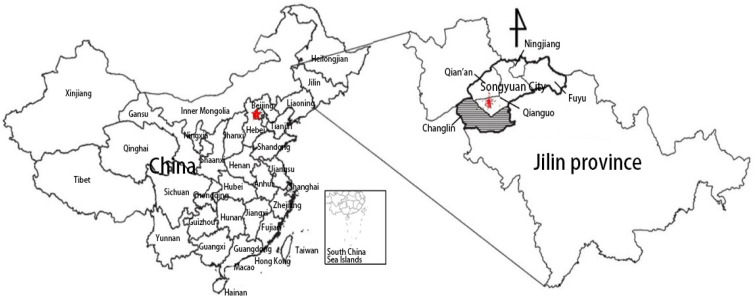
Location of study area. Note: The red spot in Jilin province indicates the epicenter of the earthquake swarm. The shaded part is the survey area.

The area of interest in this study is Songyuan (Figure 1), a city in the midwest of Jilin province, China, located at 123°6′–126°11′ E and 43°59′–45°32′ N. Songyuan is the only VIII degree high earthquake intensity zone in Jilin province, and is determined as one of the key monitoring defensive regions of China [70]. There are three tectonic lineaments in Songyuan: the northeast trending Fuyu-Zhaodong Fault; the north-west trending Second Songhua River Fault; and the north-north trending Nen River Fault [71]. Songyuan City includes Changling County; Qianguo County; Qian’an County; Fuyu County-level City; and Ningjiang District.

In 1119 A.D., a destructive earthquake of magnitude 6$\frac{3}{4}$ occurred in Songyuan City, the largest shallow-focus earthquake recorded in Northeast China to date. According to the record in the book *Big King Kingdom History*, “thousands of people died in the earthquake” [72]. In 2003, a rare earthquake swarm occurred here for the first time since Jilin province was established. On 31 March, 2006, the city was hit by a second earthquake swarm, with the maximum magnitude reaching to 5.0. On 31 October, 2013, an Ms. 5.5 earthquake occurred again in this area, and more than 200 earthquakes occurred in the following month, including more than 10 earthquakes with Ms. ≥ 4.0. In particular, Ms. 5.3, Ms. 5.8, and Ms. 5.0 earthquakes occurred in situ on 22 and 23 November, respectively [73,74], as shown in Table 3. The Ms. 5.8 earthquake was the largest earthquake in the Songyuan area since 1119 A.D. The affected population in the Songyuan area reached more than 200,000, and severe damage was caused. Thirteen people were injured [75], and more than 57,000 houses were damaged in Qianguo County, Qian’an County, and Changling County, including 16,000 houses that were severely destroyed, 40,000 houses that were slightly damaged, and nearly 310 houses collapsed [76]. The economic loss is up to CNY 20 billion [76]. The worst-hit areas were Chaganhua Town in Qianguo County and Anzi Town in Qian’an County (the area around the epicenter, red zone in Figure 1). The earthquakes occurred in winter where the extreme minimum temperature was −36.1 °C in the affected areas. As the damaged houses were unable to be repaired and rebuilt in the winter, the victims had to find accommodation with relatives and friends or rent houses in other areas to get through the winter, which meant that all the people in the two towns left the area. Therefore, this study chose Changling County as the survey area, given that it was the closest to the epicenter and therefore more representative.

### 3.2. Data Collection

#### 3.2.1. Questionnaire Design

The anonymous questionnaire in Mandarin consisted of three sections. The first section asked questions on information seeking behavior and its possible determinants (seen [3] for details). The second section surveyed the earthquake disaster information needs (information expected acquisition), information supply (information actual acquisition), and the communication channels used during daily life, earthquake emergencies, earthquake relief, and post-disaster reconstruction, which were used in this research. This section was designed according to the recent literature on earthquake information classification [20,21,22,23,24,25,26] and the basic information needs of individuals in earthquake disasters [66], and was newly developed (Supplementary File S1 with * sign). As people in risky situations tend to acquire various types of information through multiple channels, this section of the questionnaire was in the form of multiple choice. As well, a survey on the reliability of information sources and information dissemination channels was conducted. All the items were measured by a five-point Likert-type scale ranging from 1–5, with the higher scores indicating higher reliability with the statement. The third section collected the information on the socioeconomic and housing characteristics of the respondents, such as gender, age, education, income, housing ownership, housing type, and housing structures, etc., with two ways to fill in the blanks and choose the answers (seen Supplementary File S1).

#### 3.2.2. Implementation

The data were collected in Changling County in Songyuan City, China in March 2014. Considering that the sample design of the survey needed cater to different regions as well as to residents with different demographic attributes and other factors, the survey therefore started at the schools with the guardians of the students as the research target using a multi-stage random sampling method to draw the sample. The specific sampling process was divided into three stages.

The first stage was the extraction of schools. In order to increase the representativeness of the schools as much as possible, using the simple random sampling method, six representative schools (No. 1 Primary School; No. 2 Primary School; No. 3 Primary School; No. 4 Primary School; No. 1 Junior High School; and No. 3 Senior High School) were selected to participate. The six schools were all part of the school district system, leading to a group distribution of the surveys, therefore achieving wide coverage.

The second stage was the sampling of classes. One class from each grade in each elementary school and five classes from each grade in each middle and high school were randomly selected in turn in each school, sampled on a non-release back sampling basis.

The third stage was the extraction of students. A total of 15 students were selected from the sampled elementary classes using systematic sampling (including 20 students in each of the first and second grades), and 20 students were selected from the middle and high school classes using systematic sampling, for a final total of 1000 students. With permission from the selected local schools, the school students received the questionnaires as a form of homework and delivered it to one of their guardians. A total of 1000 questionnaires were sent out, and 973 were completed with a response rate of 97.3%. There were three possible reasons for this high response rate: one was the recent earthquake swarm experience, so the topic of this survey was related to the parents’ own daily lives; the second reason is the respect paid by Chinese parents to teachers’ authority (i.e., they usually follow teachers’ requests) [77]; and the third was that the students believed they would be awarded a mark for filling in the questionnaire (homework).

### 3.3. Research Indicator

The information deviation index (IDI) refers to the proportion of the difference between the expected information to be acquired (information need) and the actual information acquired (information supply) with the expected information to be acquired:

Formula: D = (E − A)/E(1)

where E represents the information expected to be acquired; A represents the information actually acquired; and D represents the information deviation index.

This means that, when the IDI value tends to 0, the information expected to be acquired and the information actually acquired is symmetrical; when the IDI value is positive, the information expected to be acquired is greater than the information actually acquired (i.e., the public’s information requirements fall short); and when the IDI value is negative, the information expected to be acquired is less than the information actually acquired (i.e., the public’s information requirements have been achieved). The greater the value of the IDI, the greater the gap between the information expected to be acquired and the information actually acquired. Conversely, the smaller the IDI value, the closer the information actually acquired is to the information expected to be acquired.

### 3.4. Analysis Techniques

#### 3.4.1. Multiple Response Analysis

Multiple response analysis, also known as polynomial analysis, has two methods: the first is the dichotomies method; and the second is category method [78]. This research adopted the dichotomies method; that is, each choice in the multiple-choice question was regarded as a variable in SPSS 16.0, and each variable was valued by 0 or 1. A value of 1 means you chose this choice, and 0 means you did not choose it. Subsequently, multiple response sets were defined, the basic variables were coded as dichotomies variable sets, and then a frequency analysis was carried out on this basis.

In this research, multiple-response analysis was used to examine the frequency (percentage) statistics of the information expected to be acquired; the information actually acquired; and the communication channels used by the public in the earthquake zone during the prodromal, acute, chronic, and crisis resolution stages of the earthquake disaster.

#### 3.4.2. Weighted Analysis

After acquiring the statistics of the frequency (percentages) of the information expected to be acquired and the information actually acquired by the public in the earthquake zone during the prodromal, acute, chronic, and crisis resolution stages of the earthquake disaster, a weighted analysis of each data was needed to facilitate the calculation of IDI in each stage.

## 4. Results

### 4.1. Socioeconomic and Housing Characteristics of Respondents

Among the 973 returned questionnaires, 918 questionnaires offered valid responses. Males were slightly over-represented (52.1%). Approximately 90% of survey participants were between the ages of 31 and 50. Approximately half of them had a high school degree or above, and over 90% of respondents earned less than CNY 100,000 per family per year. More than 80% owned their own house, with bungalows (43.9%) and multilayer buildings (47.7%) as the main housing styles, with masonry-concrete structures (52.4%) and reinforced-concrete structures (44%) as the main housing structures.

### 4.2. Information Content Preferences of Earthquake Disaster

#### 4.2.1. The Prodromal Stage of an Earthquake Disaster

1. The information expected to be acquired

**Figure 2:**
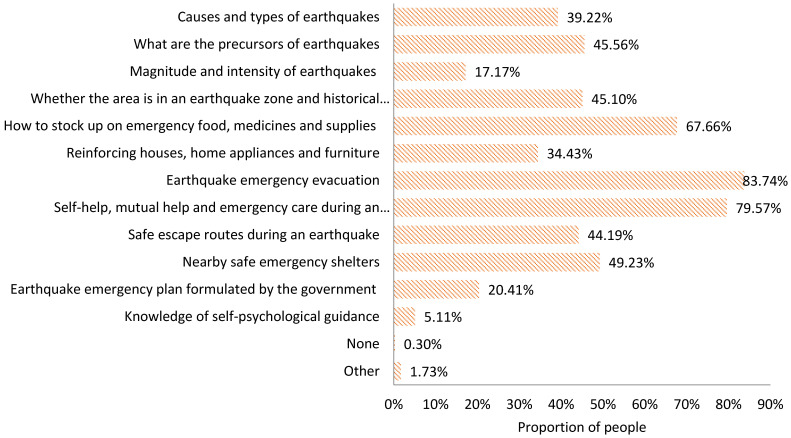
Information expected to be acquired by the public in the prodromal stage of an earthquake disaster.

In daily life, when seeking earthquake risk knowledge, the public expects to acquire the following types of information (Figure 2):

- A basic knowledge of earthquakes, including knowledge regarding the causes and types of earthquakes (39.22%), what the precursors of earthquakes are (45.56%), and information about the magnitude and intensity of earthquakes (17.17%);
- Earthquake preparedness knowledge, including whether the local area is in an earthquake zone and the historical earthquake occurrences (45.10%), guidance regarding of how to stock up on emergency food, medicines and supplies (67.66%), and information about reinforcing houses, home appliances, and furniture (34.43%);
- Earthquake emergency response knowledge, including information about earthquake emergency evacuation (83.74%), self–help, mutual help and emergency care information during an earthquake (79.57%), safe escape route guidance during an earthquake (44.19%), and details about nearby safe emergency shelter (49.23%);
- The earthquake emergency plan formulated by the government (20.41%);
- Self-psychological guidance knowledge (5.11%).

2. The information actually acquired

**Figure 3:**
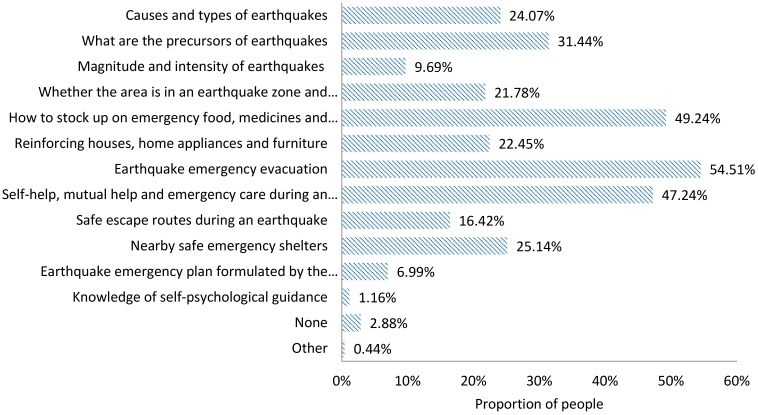
Information actually acquired by the public in the prodromal stage of an earthquake disaster.

In daily life, the types of earthquake information actually acquired by the public in daily life are ranked as follows (Figure 3):

Information about earthquake emergency evacuation (54.51%); guidance regarding how to stock up on emergency food, medicine and supplies (49.24%); self–help, mutual help and emergency care information during an earthquake (47.24%); what the precursors of earthquakes are (31.44%); details about nearby safe emergency shelter (25.14%); knowledge regarding the causes and types of earthquakes (24.07%); information about reinforcing houses, home appliances and furniture (22.45%); whether the local area is in an earthquake zone and the historical earthquakes occurrences (21.78%); safe escape route guidance during an earthquake (16.42%); information about the magnitude and intensity of earthquakes (9.69%); the earthquake emergency plans formulated by the government (6.99%); and self-psychological guidance knowledge (1.16%).

3. Information deviation index

According to the definition of IDI above, it can be seen that, in daily life, the deviation between the information expected and the information actually acquired by the people in the disaster zone presents different characteristics (Table 4).

The IDI values for self-help, mutual help, and emergency care information during an earthquake and knowledge regarding the causes and types of earthquakes are −0.01 and −0.04, respectively, both of which are negative values, indicating that the public’s expected information on these two indicators is satisfied. The two values tend to be close to 0, representing that the actual information acquired is close to the expected information acquired. This indicates that the information the people want and the information they finally get are symmetrical. In addition, the IDI values for information about earthquake emergency evacuation; information about reinforcing houses, home appliances, and furniture; what the precursors of earthquakes are; and guidance regarding how to stock up on emergency food, medicines, and supplies are −0.11, −0.11, −0.17, and −0.24, respectively, indicating that the actual information that can be acquired about these four indicators has exceeded the information that people expect to acquire, and that people’s expectations can be met. The IDI value of 0.04 for the knowledge regarding the earthquake magnitude and intensity is positive, indicating that the information expected by the public for this indicator has not been met, but the result tends to be close to 0, indicating that the actual information acquired is close to the expected information. The IDI values for details about nearby safe evacuation sites, whether the local area is in the earthquake zone and the historical earthquake occurrences, safe escape route guidance during an earthquake, the earthquake emergency plans formulated by the government, and knowledge of self-psychological guidance are 0.13, 0.18, 0.37, 0.42, and 0.61, respectively, indicating that people’s expectations of acquiring the information relating to these five aspects are not satisfied.

#### 4.2.2. The Acute Stage of an Earthquake Disaster

**Figure 4:**
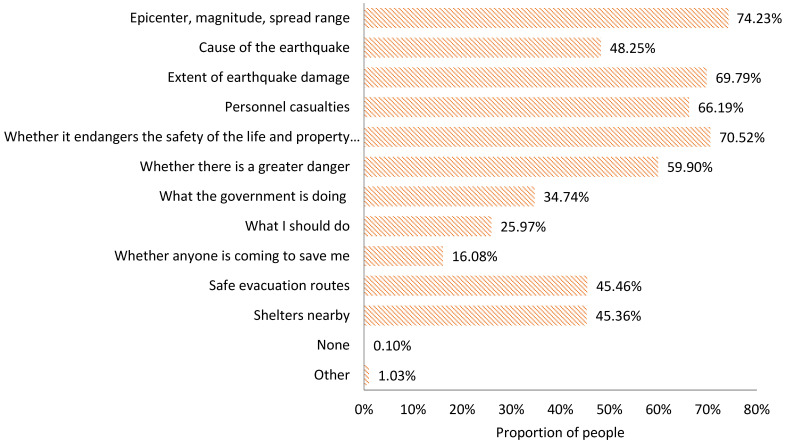
Information expected to be acquired by the public in the acute stage of an earthquake disaster.

1. The information expected to be acquired (Figure 4) is as follows:

- Earthquake information, including the epicenter, magnitude, and spread range (74.23%), and cause of the earthquake (48.25%);
- Disaster information, including the extent of earthquake damage (69.79%) and personnel casualties (66.19%);
- Public psychological reaction information, including whether it endangers the safety of the life and property of oneself or relatives and friends (70.52%), whether there is a greater danger (59.90%), what the government is doing (34.74%), what I should do (25.97%), and whether anyone is coming to save me (16.08%);
- Emergency evacuation and shelter information, including safe evacuation routes (45.46%) and shelters nearby (45.36%).

2. The information actually acquired

**Figure 5:**
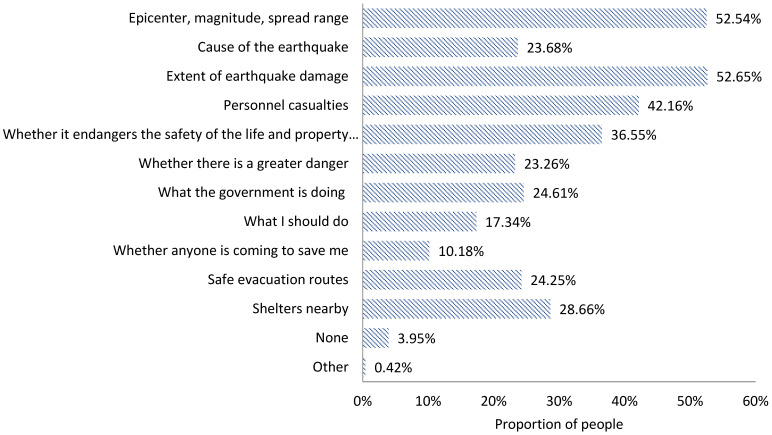
Information actually acquired by the public in the acute stage of an earthquake disaster.

At the first time of the earthquake, the ranking of the earthquake-related information actually acquired by the public is in order (Figure 5):

The extent of earthquake damage (52.65%); and earthquake epicenter, magnitude, and spread range (52.54%); personnel casualties (42.16%); whether it endangers the safety of the life and property of oneself or relatives and friends (36.55%); shelters nearby (28.66%); what the government is doing (24.61%); safe evacuation routes (24.25%); causes of the earthquake (23.68%); whether there is a greater danger (23.26%); what I should do (17.34%); and whether anyone is coming to save me (10.18%).

3. Information deviation index

According to the definition of IDI above, it can be seen that, in the first period after an earthquake, the deviation between the information expected by the people in the disaster area and the information actually acquired presents different characteristics (Table 5). 

The IDI value regarding safe evacuation routes is 0, which means that the information people want to receive about safe evacuation routes and the information they finally receive are symmetrical. The IDI values regarding shelters nearby, whether anyone is coming to save me, personnel casualties, and what I should do are −0.02, −0.02, −0.03, and −0.08, respectively, which are negative values, indicating that people’s expectations of information about these four indicators are met. An IDI value tending to be 0 proves that the actual information is close to the expectation. In addition, the IDI value concerning the extent of earthquake damage, the information on the magnitude, and extent of the earthquake and the information on what the government is doing are −0.22, −0.15, and −0.15, respectively, indicating that people’s expectations about these three indicators can be met. The IDI values with regard to whether there is a greater danger and the cause of the earthquake are 0.37 and 0.20, respectively, indicating that people’s expectations of acquiring information about these two aspects have not been met.

#### 4.2.3. The Chronic Stage of an Earthquake Disaster

1. The information expected to be acquired

**Figure 6:**
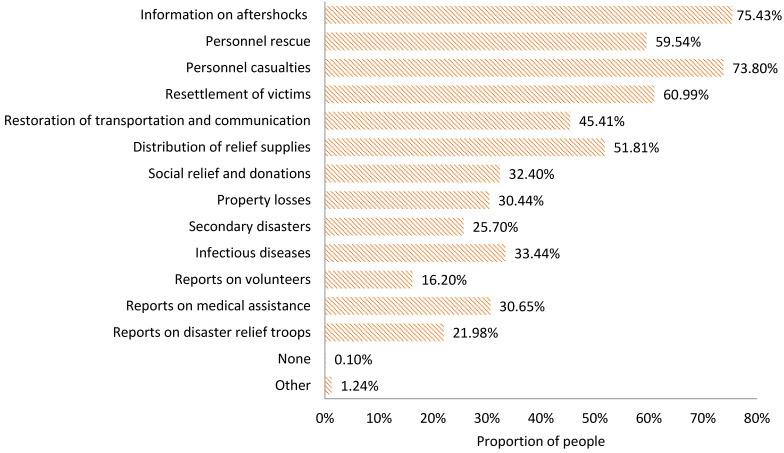
Information expected to be acquired by the public in the chronic stage of an earthquake disaster.

During the earthquake relief period, the earthquake disaster information that people in the disaster area expect to acquire is as follows (Figure 6):

- Earthquake information, including information on aftershocks (75.43%);
- Disaster information, including personnel casualties (73.80%), property losses (30.44%), secondary disasters (25.70%), and infectious diseases (33.44%);
- Real-time progress information on disaster relief and rescue, including personnel rescue (59.54%), the resettlement of victims (60.99%), and the restoration of transportation and communication (45.41%);
- Relief supplies and donations information, including the distribution of relief supplies (51.81%) and social relief and donations (32.40%);
- Domestic and foreign media reported information, including reports on volunteers (16.20%), medical assistance (30.65%), and disaster relief troops (21.98%).

2. The information actually acquired

**Figure 7:**
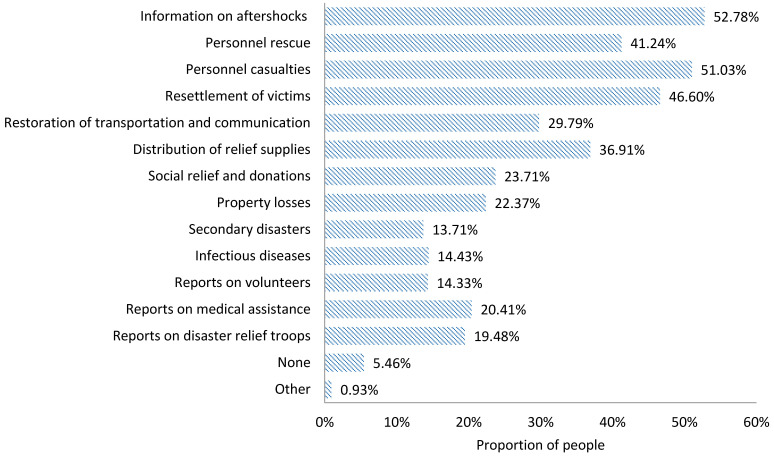
Information actually acquired by the public in the chronic stage of an earthquake disaster.

In the earthquake relief period, the information actually acquired by the victims is ranked as follows (Figure 7): information on aftershocks (52.78%), personnel casualties (51.03%), resettlement of victims (46.60%), personnel rescue (41.24%), distribution of relief supplies (36.91%), the restoration of transportation and communication (29.79%), social relief and donations (23.71%), property losses (22.37%), reports on medical assistance (20.41%), reports on disaster relief troops (19.48%), infectious diseases (14.43%), reports on volunteers (14.33%), and secondary disasters (13.71%).

3. Information deviation index

According to the IDI definition above, it can be seen that, during the earthquake relief period, the deviation between the information expected by the people in the disaster area and the information actually acquired shows different characteristics (Table 6). 

The IDI value of information on aftershocks is 0, which shows that the information people want regarding aftershocks and the information they finally receive are symmetrical. The IDI values of personnel rescue, personnel casualties, reports on medical assistance, and the restoration of transportation and communication are 0.02, 0.02, 0.06, and 0.07, respectively, which are all positive, indicating that people’s expected information about these four indicators is not satisfied. However, the results tend to be close to 0, indicating that the information actually acquired is close to the expected information. The IDI values of information on the distribution of relief supplies, social relief and donations, property losses, and the resettlement of victims are −0.01, −0.04, −0.05, and −0.09, respectively, which are all negative, indicating that people’s expected information on these four indicators is satisfied and the result is close to 0, also indicating that the actual information acquired is close to the expected information acquired. In addition, the IDI values of secondary disasters and infectious diseases are 0.24 and 0.39, respectively, indicating that the public’s expectation of acquiring relevant information about these two aspects has not been met. The IDI values of reports on volunteers and disaster relief troops are −0.26 and−0.26, indicating that the information available on these two aspects has exceeded the expectations of the public, and that the expectations of the public can be met. 

#### 4.2.4. The Resolution Stage of an Earthquake Disaster

1. The information expected to be acquired

**Figure 8:**
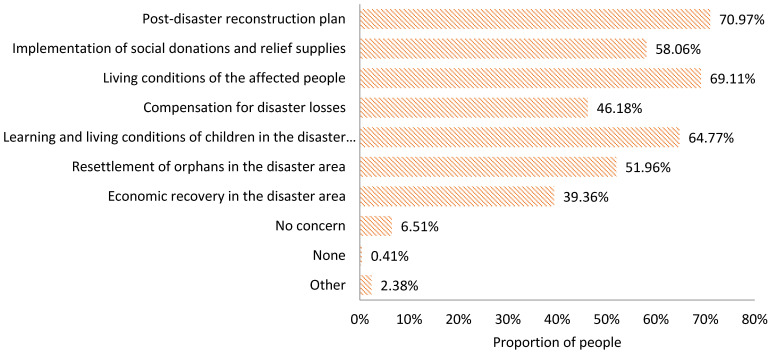
Information expected to be acquired by the public in the resolution stage of an earthquake disaster.

During the post-disaster reconstruction period, the information that people in the disaster area expect to acquire is divided into the following categories (Figure 8):

- Post-disaster reconstruction plan (70.97%);
- Implementation of social donations and relief supplies (58.06%);
- The situation of disaster victims, including the living conditions of the affected people (69.11%), the compensation for disaster losses (46.18%), the learning and living conditions of children in the disaster area (64.77%), and the resettlement of orphans in the disaster area (51.96%);
- The situation in the disaster area, including the economic recovery in the disaster area (39.36%).

2. The information actually acquired

**Figure 9:**
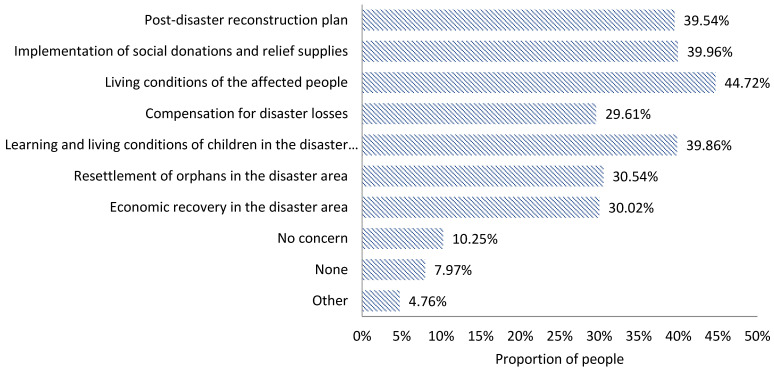
Information actually acquired by the public in the resolution stage of an earthquake disaster.

During the post-disaster reconstruction period, the actual information acquired by the people in the disaster area is sorted as follows (Figure 9): the living conditions of the affected people (44.72%), the implementation of social donations and relief supplies (39.96%), the learning and living conditions of children in the disaster-hit areas (39.86%), the post-disaster reconstruction plan (39.54%), the resettlement of orphans in the disaster area (30.54%), the economic recovery in the disaster area (30.02%), and the compensation for disaster losses (29.61%).

3. Information deviation index

According to the above definition of IDI, it can be seen that, during the earthquake disaster recovery period, the deviation between the expected information and the actual information acquired by the people in disaster areas presents different characteristics (Table 7).

The information deviation index values of the information regarding the living conditions of the affected people, the compensation for disaster losses, and the learning and living conditions of children in the affected area are 0.04, 0.05, and 0.09, respectively, all of which are positive, indicating that people’s expected information regarding these three indicators could not be satisfied; however, the results tend to be close to 0, suggesting that the actually acquired information is close to the expected information. The IDI value of the information on the implementation of social donations and disaster relief supplies is −0.02 and negative, indicating that people’s expectations of the indicator has been met and approach 0, indicating that the actual information acquired is close to the expected information. In addition, the IDI values of the post-disaster reconstruction plan and the resettlement of orphans in the disaster areas are 0.18 and 0.13, respectively, indicating that people’s expectations of acquiring relevant information on these two aspects have not been met. Moreover, the IDI value of information about the economic recovery in the disaster area is −0.13, which indicates that the information about this area is actually more available than people expect; thus, the people’s expectations can be met.

#### 4.2.5. Main Risk Information Composition of in Different Stages of an Earthquake Disaster

**Figure 10:**
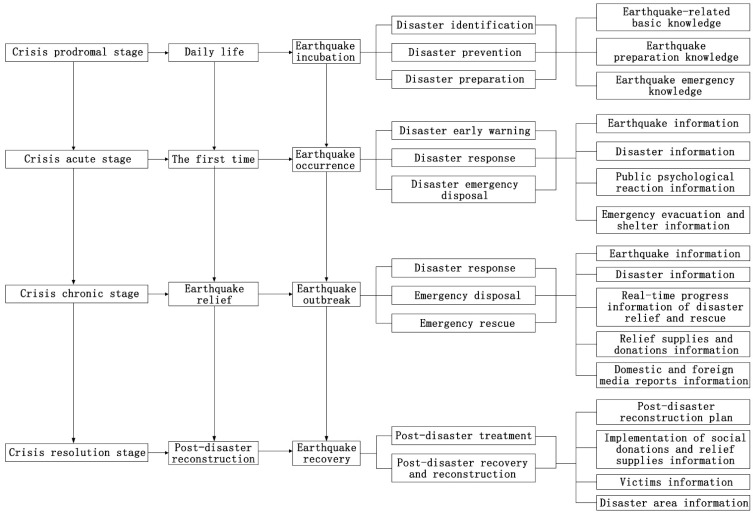
Main information composition in the different stages of an earthquake disaster.

Based on the above research, the composition of the main risk information on earthquake disasters is in different stages, as shown in Figure 10. In other words, in the prodromal stage of an earthquake disaster, earthquake risk management should focus on disaster identification, disaster prevention, and disaster preparation. At this time, the main risk information should include earthquake-related basic knowledge, earthquake preparation knowledge, and earthquake emergency knowledge. In the acute stage of an earthquake disaster, earthquake risk management should focus on disaster early warning, disaster response, and emergency disposal, while the risk information released should mainly include earthquake information, disaster information, public psychological reaction information, and emergency evacuation and shelter information. In the chronic stage of an earthquake disaster, earthquake risk management should focus on disaster response, emergency disposal, and emergency rescue, while the risk information at this time should mainly be earthquake information, disaster information, real-time progress information on disaster relief and rescue, relief supplies and donations information, and domestic and foreign media information. In the recovery stage of an earthquake disaster, earthquake risk management should mainly be focused on the post-disaster treatment and post-disaster recovery and reconstruction, while the risk information at this time should focus on the post-disaster reconstruction plan, information about the implementation of social donations and relief supplies, information about the victims, and information about the disaster area.

### 4.3. Information Communication Channel Preferences of Earthquake Disaster

#### 4.3.1. The Prodromal Stage of an Earthquake Disaster

In daily life, 74.22% of the respondents acquire earthquake-related knowledge and information through TV reports, which indicates that TV, as a traditional media, still plays a pivotal role in the dissemination of earthquake knowledge and information. The second most common channel is the internet, accounting for 53.76%, which also illustrates the indispensable role of the internet as a new medium in risk communication. The third most common channel is interpersonal communication, with 40.81% of respondents believing that interpersonal communication is the easiest and fastest way to acquire earthquake disaster information and knowledge. Radio broadcasts (33.19%), newspapers and magazines (20.04%), and mobile phone text messages (19.73%) also proved to be effective means of communication (shown in Table 8).

#### 4.3.2. The Acute Stage of an Earthquake Disaster

At the first time of the earthquake, people in the earthquake area have obvious preference when they choose information acquisition channels. The usage rates of different channels vary greatly, with more than 60% of people still preferring TV media (61.24%) as a channel to acquire earthquake-related information, and more than 40% of people choosing the internet (42.99%). While fewer people choose newspapers and magazines (10.10%), probably because newspapers and magazines have a long production cycles and weak timeliness, and when an earthquake has just occurred, people urgently need to know about the earthquake, so fewer people choose newspapers and magazines to get information. People who choose interpersonal communication, mobile phone text messages, and radio broadcasts account for 24.33%, 23.00%, and 17.32%, respectively (as shown in Table 8).

#### 4.3.3. The Chronic Stage of an Earthquake Disaster

During the earthquake relief period, TV, as the “first media”, still reflects its important position and role in information dissemination, accounting for 55.77%. Moreover, the internet, for its convenience and strong interactivity, has also become the information communication medium and information acquisition channel relied on by the public, accounting for 41.44%. Radio broadcasts, interpersonal communication, and mobile phone text messages play similar roles in the spreading period of earthquake disaster, accounting for 22.47%, 21.34%, and 20.62%, respectively, while newspapers and magazines are the lowest, accounting for 17.73% (shown in Table 8).

#### 4.3.4. The Resolution Stage of an Earthquake Disaster

During the post-disaster reconstruction period, more than half of the respondents still choose to use television (55.28%) to acquire earthquake-related information, followed closely by media, such as the internet (42.55%), interpersonal communication (23.19%), radio broadcasts (19.36%), newspapers and magazines (16.77%), and mobile phone text messages (16.15%) (shown in Table 8).

## 5. Discussion

There were two objectives in this research. The first focused on individuals’ information needs and communication preferences. Two questions were examined concerning what information content is needed and how this information should be disseminated.

With regard to the “what” question, descriptive statistics revealed that individuals’ information needs are highly differentiated at the different stages of an earthquake. Before an earthquake occurrence, individuals are mainly concerned with earthquake disaster preparedness and response knowledge. For example, the top three expected information are knowledge about earthquake emergency evacuation; knowledge regarding self-help, mutual help, and emergency care during an earthquake; and knowledge of how to stock up on emergency food, medicines, and supplies. In the first period after an earthquake occurrence, it can be seen that the public is eager to acquire basic information related to the earthquake disaster itself and information related to their own interests, which is consistent with the results of previous studies [30,66]. It can also be seen that, during an earthquake relief period, people in the earthquake zone are firstly concerned about earthquake information and disaster information, and then concerned about real-time progress information on disaster relief and rescue, which confirms previous reports [30,66]. During an earthquake disaster recovery period, the public in the earthquake zone is most concerned with the post-disaster reconstruction plan formulated by the government, which is consistent with previous study results [30,66]. Evidently, individuals show a shift in information needs from “preparedness and response knowledge” to “disaster information”, then to “disaster information and disaster relief information” in parallel, to “reconstruction and reflection information”.

In addition, as mentioned above, previous studies on earthquake emergency disaster information classification are missing information classification for the period before an earthquake; therefore, this has been added to this study and divided into basic earthquake knowledge, preparedness knowledge, and emergency response knowledge. Moreover, earthquake damage information in previous studies [20,21,22,23,24] mainly focused on information regarding the direct damage caused by earthquakes, and neglected information on the indirect damage caused by earthquakes (i.e., the psychological impact of earthquakes on the public), which this study includes.

Regarding the “how” question, respondents were asked about previously used and preferred communication channels for the dissemination of earthquake information. As indicated in previous studies [26,42,43,44], the TV and the internet are the most effective channels. However, the range of internet channels is very wide, and may include search engines, forums, posting, social media, etc. This paper does not make a distinction between these internet channels, which may be a topic for a future in-depth study, particularly the use of social media. In addition, smartphone apps [44,79,80,81] have now become an important information dissemination channel. These apps are not explored in this paper but may be studied in depth in the future.

In addition to the “what” question and the “how” question, there is another question that needs to be answered—the “when” question, i.e., when this information should be disseminated. This is another area for future research.

With regard to the second research objective, an information deviation index indicator was developed to evaluate the difference between information need and information supply. We used the information expected to be acquired as a representative of information need, and used the information actually acquired as a representative of information supply. There may be room for a certain amount of error, especially between the information supply and the information actually acquired.

In addition to the research objectives, the data acquisition method is also worth discussing. The data of this study were obtained from field investigation. Field investigation revealed disaster information needs in detail, but the timeliness and regional range of acquiring disaster information were limited [82]. Future methods of acquiring data may consider combining field investigation with big data mining, such as using data extracted from social media [24,83,84,85,86,87,88,89,90,91], Google Trends [92,93], and Baidu Index [94,95]. In particular, these several data sources can make it possible to quite easily track the behavior of Internet users in real time. For example, disaster managers can benefit from these tools in the “acute stage” to plan targeted information and communication campaigns, thus increasing their usefulness.

## 6. Conclusions

This research investigated the information content and communication channel preferences from individuals’ needs perspective within the setting of earthquake disaster in Chinese society. Compared to a survey of respondents of a place only potentially vulnerable to a risk, the survey presented to the victims of an earthquake stricken area, and provided more realistic and persuasive results. Next, it proposed the main composition of information at the different stages of an earthquake disaster, and clarified which types of earthquake disaster risk information should be given priority at each stage. Moreover, this research developed an IDI to evaluate the efficiency of information supply at each stage based on whether the respondents’ information needs are being met, which made up the assessment method of risk communication effectiveness. The research can be replicated in other areas subject to seismic risk, and the policymakers can benefit from the results of this research, as the research draws the following guidance on earthquake risk management.

1. In the prodromal stage, risk management should give priority to the identification, prevention and preparation for earthquake disaster. First, risk management efforts should publicize and popularize knowledge of safety precautions (including basic earthquake-related knowledge and emergency knowledge), help people to establish crisis concepts, mitigate their sense of hardship, assist people to master the skills of self-help and mutual help when faced with an earthquake, and improve their ability to cope with a crisis. Second, risk management work should establish and improve the early warning systems for earthquake disaster, and prepare the public for upcoming earthquake crises; thus, the risk information should include knowledge about preparedness. Third, risk management tasks should include preparing a good earthquake emergency plan in advance.
2. In the acute stage, risk management should focus on early warning, response, and emergency disposal. First, risk communication should include releasing earthquake disaster early warning information to the public in a timely fashion as accurately as possible. Second, risk communication should include an overview of the earthquake, the disaster and its development trend information. Third, risk communication should involve the release of emergency disposal information, such as public psychological reaction information, and emergency evacuation and shelter information.
3. In the chronic stage, earthquake risk management should focus on disaster response, emergency disposal, and emergency rescue. First, earthquake and disaster information should be reported to the public in a timely and comprehensive manner. Second, real-time progress information on disaster relief and rescue, and information on relief supplies and donations should be accurate and objective. Third, risk communication should concentrate on the domestic and foreign media reported information, particularly the reports on volunteers, medical assistance, and disaster relief troops. These positive reports can inspire people’s morale and boost their confidence. Fourth, pamphlets should be prepared to help the public with guidance on psychological and adjustment.
4. In the resolution stage, earthquake risk management should concentrate on post-disaster treatment and post-disaster recovery and reconstruction, while encouraging people to be self-reliant, overcome difficulties, rebuild scientifically, restore normal production and living order, and eliminate the impact of the crisis. Therefore, risk information at this time should focus on the post-disaster reconstruction plan, information about the implementation of social donations and relief supplies, information about the victims, and information about the disaster area.
5. Using the IDI to evaluate the information supply by official information sources at each stage, we found that most of the public’s information needs are met, with a few exceptions. Indicators such as, in daily life, information about safe evacuation sites nearby, safe escape routes during an earthquake, the earthquake emergency plans formulated by the government, and knowledge of psychological guidance are not met. This suggests that the governments should include these categories when releasing information in the future.
6. Through an analysis of the communication channel preferences for earthquake disaster information at each stage, it is concluded that the two most prominent information acquisition channels are television and the internet, with radio broadcasts, interpersonal communication, newspapers and magazines, and mobile phone SMS also being effective communication channels. Therefore, when releasing earthquake disaster information in the future, it is recommended that the priority dissemination channels should be the TV and the internet.

In viewing these results, some limitations of this research should be explained.

(a) The study area was an earthquake-stricken area. Further research should add non-earthquake areas as the study area to compare whether there are differences between the two areas regarding the residents’ earthquake disaster information preferences.

(b) The elderly and younger social groups were missing from the respondents group in this study, and these groups are very vulnerable during such disasters. In this research, 3% of respondents were aged over 60 years, and there were no respondents below the age of 14. To date, few studies have investigated the risk information needs of the elderly and the young; this should be an area of interest for future research. 

(c) This information preference research focuses on information content and communication channel preferences. In future, the study of information expression could be deepened by exploring the accessibility and ease of use of information, i.e., the type of information presentation formats (e.g., text, image, video) used by people to access and utilize information.

## Figures and Tables

**Table 1:** Earthquake emergency disaster information classification.

Scholars	Earthquake Emergency Information Classification
Nie et al. [18]	Maps; socio-economic statistics; earthquake basic data; engineering earthquake data; disaster influence background; disaster-related factors; disaster relief force reserve; earthquake emergency contact; earthquake emergency plan.
Su et al. [19]	Group of the basic background; earthquake disasters and disaster relieving background; law and regulations; the EER prearranged scheme and disaster reduction planning; existing earthquake disaster countermeasures; experience from earthquake disaster reduction demonstrations and maneuvers; seismic station networks; emergency communications; historical earthquakes; earthquake disaster relieve cases; disaster relieve capacity reserve conditions; earthquake hazards; earthquake disasters; emergency decision-making and its assistant knowledge; disaster field status; allotment and deployment information of the disaster relieve materials and personnel; social response circumstances.
Bai et al. [20]	Essential earthquake information; damage information; emergency response information; disposal efficiency information.
Zhu et al. [21]	Seismic conditions; casualties; housing damage; secondary disasters; damage to transportation systems; damage to water supply systems; damage to power supply systems; damage to gas supply systems; damage to communication systems.
Dong et al. [22]	Seismic information; background information of earthquake zone; disaster information; emergency response.
Zhang et al. [23]	earthquake information; disaster information; earthquake area background information; emergency response information; rescue information; popular science information.
Su et al. [24]	Location information; time information; disaster investigation; social public opinion; emergency rescue; emergency support; non-emergency; comprehensive classes.
Sapountzaki [25] Fokaefs [26]	Informative messages; warning messages; consulting messages; guiding messages

**Table 2:** Periodicity and the characteristics of the stages of crisis events.

Stages	Characteristics	Actions
Crisis Prodromal Stage	a. The result of contradiction accumulation;	a. Crisis identification
	b. Unnoticeable;	b. Crisis prevention
	c. Easiest stage of crisis management.	c. Crisis preparation
Crisis Acute Stage	a. The shortest duration but the longest feeling;	a. Crisis early warming
	b. Greatest damage to society;	b. Crisis response
	c. High social attention.	c. Crisis emergency disposal
Crisis Chronic Stage	a. Long duration;	a. Crisis response
	b. Crisis management helps to shorten the duration;	b. Emergency disposal
	c. Crisis losses have been caused.	c. Emergency rescue
Crisis Resolution Stage	a. Complete relief from crisis impact;	a. Post-disaster treatment
	b. Social attention turns to eliminate event impact;	b. Post-disaster recovery and reconstruction
	c. Linked to the next prodromal stage.	

**Table 3:** Basic seismic parameters of Songyuan Ms. ≥ 5.0 earthquake swarm in 2013 [73,74].

Serial Number	Earthquake Time (GMT+8)	Latitude (°)	Longitude (°)	Focal Depth (km)	Magnitude
1	31 October 2013 11:03	44.60	124.18	8	5.5
2	31 October 2013 11:10	44.60	124.20	6	5.0
3	22 November 2013 16:18	44.72	124.14	8	5.3
4	23 November 2013 06:04	44.60	124.10	9	5.8
5	23 November 2013 06:32	44.60	124.10	8	5.0

**Table 4:** Information deviation index in the prodromal stage of an earthquake disaster.

Types	Index	Expected Information before Weighting	Expected Information after Weighting	Actual Information before Weighting	Actual Information after Weighting	IDI	Information Content ^1^
Basic earthquake knowledge	Causes and types of earthquakes	39.22%	7.35%	24.07%	7.68%	−0.04	√
	What are the precursors of earthquakes	45.56%	8.54%	31.44%	10.03%	−0.17	√
	Magnitude and intensity of earthquakes	17.17%	3.22%	9.69%	3.09%	0.04	×
Earthquake preparedness knowledge	Whether the local area is in an earthquake zone and the historical earthquake occurrences	45.10%	8.45%	21.78%	6.95%	0.18	×
	How to stock up on emergency food, medicines and supplies	67.66%	12.68%	49.24%	15.71%	−0.24	√
	Reinforcing houses, home appliances and furniture	34.43%	6.45%	22.45%	7.16%	−0.11	√
Earthquake emergency response knowledge	Earthquake emergency evacuation	83.74%	15.70%	54.51%	17.39%	−0.11	√
	Self–help, mutual help and emergency care	79.57%	14.92%	47.24%	15.07%	−0.01	√
	Safe escape routes during an earthquake	44.19%	8.28%	16.42%	5.24%	0.37	×
	Nearby safe emergency shelter	49.23%	9.23%	25.14%	8.02%	0.13	×
——	Earthquake emergency plan formulated by the government	20.41%	3.83%	6.99%	2.23%	0.42	×
	Self-psychological guidance	5.11%	0.96%	1.16%	0.37%	0.61	×
	None	0.30%	0.06%	2.88%	0.92%	−14.33	√
	Other	1.73%	0.32%	0.44%	0.14%	0.56	×
	Total	533.42%	100.00%	313.45%	100.00%	——	——

^1^ √ represents satisfaction; × means unsatisfactory.

**Table 5:** Information deviation index in the acute stage of an earthquake disaster.

Types	Index	Expected Information before Weighting	Expected Information after Weighting	Actual Information before Weighting	Actual Information after Weighting	IDI	Information Content ^1^
Earthquake information	The epicenter, magnitude, spread range of the earthquake	74.23%	13.31%	52.54%	15.25%	−0.15	√
	The cause of the earthquake	48.25%	8.64%	23.68%	6.89%	0.20	×
Disaster information	The extent of earthquake damage	69.79%	12.52%	52.65%	15.28%	−0.22	√
	Personnel casualties	66.19%	11.87%	42.16%	12.26%	−0.03	√
Public psychological response information	Whether it endangers the safety of the life and property safety of oneself or relatives and friends	70.52%	12.64%	36.55%	10.63%	0.16	×
	Whether there is a greater danger	59.90%	10.74%	23.26%	6.77%	0.37	×
	What the government is doing	34.74%	6.22%	24.61%	7.15%	−0.15	√
	What I should do	25.97%	4.66%	17.34%	5.03%	−0.08	√
	Whether anyone is coming to save me	16.08%	2.89%	10.18%	2.96%	−0.02	√
Emergency evacuation and shelter information	Safe evacuation routes	20.41%	3.83%	6.99%	2.23%	0.42	×
	Shelters nearby	5.11%	0.96%	1.16%	0.37%	0.61	×
——	None	0.10%	0.02%	3.95%	1.13%	−55.50	√
	Other	1.03%	0.18%	0.42%	0.12%	0.33	×
	Total	557.60%	100.00%	340.25%	100.00%	——	——

^1^ √ represents satisfaction; × means unsatisfactory.

**Table 6:** Information deviation index in the chronic stage of an earthquake disaster.

Types	Index	Expected Information before Weighting	Expected Information after Weighting	Actual Information before Weighting	Actual Information after Weighting	IDI	Information Content ^1^
Earthquake information	Information on aftershocks	75.43%	13.49%	52.78%	13.42%	0.00	√
Disaster information	Personnel casualties	73.80%	13.20%	51.03%	12.98%	0.02	×
	Property losses	30.44%	5.44%	22.37%	5.69%	−0.05	√
	Secondary disasters	25.70%	4.60%	13.71%	3.49%	0.24	×
	Infectious diseases	33.44%	5.98%	14.43%	3.67%	0.39	×
Real-time progress information on disaster relief and rescue	Personnel rescue	59.54%	10.65%	41.24%	10.49%	0.02	×
	Resettlement of victims	60.99%	10.91%	46.60%	11.85%	−0.09	√
	The restoration of transportation and communication	45.41%	8.12%	29.79%	7.58%	0.07	×
Relief supplies and donations information	Distribution of relief supplies	51.81%	9.27%	36.91%	9.39%	−0.01	√
	Social relief and donations	32.40%	5.80%	23.71%	6.03%	−0.04	√
Domestic and foreign media reported information	Reports on volunteers	16.20%	2.90%	14.33%	3.64%	−0.26	√
	Reports on medical assistance	30.65%	5.48%	20.41%	5.19%	0.06	×
	Reports on disaster relief troops	21.98%	3.93%	19.48%	4.95%	−0.26	√
——	None	0.10%	0.02%	5.46%	1.39%	−74.23	√
	Other	1.24%	0.22%	0.93%	0.24%	−0.07	√
	Total	559.13%	100.00%	393.20%	100.00%	——	——

^1^ √ represents satisfaction; × means unsatisfactory.

**Table 7:** Information deviation index in the resolution stage of an earthquake disaster.

Index	Expected Information before Weighting	Expected Information after Weighting	Actual Information before Weighting	Actual Information after Weighting	IDI	Information Content ^1^
Post-disaster reconstruction plan	70.97%	17.32%	39.54%	14.26%	0.18	×
Implementation of social donations and relief supplies	58.06%	14.17%	39.96%	14.41%	−0.02	√
Living conditions of the affected people	69.11%	16.87%	44.72%	16.13%	0.04	×
The compensation for disaster losses	46.18%	11.27%	29.61%	10.68%	0.05	×
The learning and living conditions of children in the disaster area	64.77%	15.81%	39.86%	14.38%	0.09	×
The resettlement of orphans in the disaster area	51.96%	12.68%	30.54%	11.02%	0.13	×
The economic recovery in the disaster area	39.36%	9.61%	30.02%	10.83%	−0.13	√
No concern	6.51%	1.59%	10.25%	3.70%	−1.33	√
None	0.41%	0.10%	7.97%	2.87%	−27.73	√
Other	2.38%	0.58%	4.76%	1.72%	−1.96	√
Total	409.71%	100.00%	277.23%	100.00%	——	——

^1^ √ represents satisfaction; × means unsatisfactory.

**Table 8:** Information communication channel preferences in the different stages of an earthquake disaster.

Types	Prodromal Stage before Weighting	Prodromal Stage after Weighting	Acute Stage before Weighting	Acute Stage after Weighting	Chronic Stage before Weighting	Chronic Stage after Weighting	Resolution Stage before Weighting	Resolution Stage after Weighting
TV	74.22%	30.57%	61.24%	33.48%	55.77%	30.07%	55.28%	30.07%
Radio broadcasts	33.19%	13.67%	17.32%	9.47%	22.47%	12.12%	19.36%	12.12%
Newspapers and magazines	20.04%	8.26%	10.10%	5.52%	17.73%	9.56%	16.77%	9.56%
The Internet	53.76%	22.14%	42.99%	23.50%	41.44%	22.34%	42.55%	22.34%
Mobile phone text messages	19.73%	8.13%	23%	12.58%	20.62%	11.12%	16.15%	11.12%
Interpersonal communication	40.81%	16.81%	24.33%	13.30%	21.34%	11.51%	23.19%	11.51%
None	1.04%	0.43%	0.93%	0.51%	2.58%	1.39%	1.14%	1.39%
Other	0%	0.00%	2.99%	1.63%	3.51%	1.89%	3.93%	1.89%
Total	242.79%	100.00%	182.90%	100.00%	185.46%	100.00%	178.37%	100.00%

## Data Availability

All data sources are fully disclosed in the body of the manuscript.

## Supplementary Materials

The following are available online at https://www.mdpi.com/article/10.3390/ijerph182413070/s1, Supplementary File S1: Survey on earthquake disaster information content and acquisition channels.
