# Supplementary material for: Information Preference and Information Supply Efficiency Evaluation before, during, and after an Earthquake: Evidence from Songyuan, China
Source: Int J Environ Res Public Health. 2021 Dec 11;18(24):13070. doi: 10.3390/ijerph182413070 (PMC8701310; doi:10.3390/ijerph182413070)
Supplement: Supplementary file 1 [file ijerph-18-13070-s001.zip › ijerph-1433459-supplementary.pdf]

**Supplementary File S1:** Questionnaire

**Section II. Survey on earthquake disaster information content and acquisition channels.**

**Q2-1 What types of information do you expect to acquire in daily life about earthquake disaster?**
**Q2-2 What types of information do you actually acquire in daily life about earthquake disaster?**

1. knowledge regarding the causes and types of earthquakes;
2. what the precursors of earthquakes are;
3. knowledge of the magnitude and intensity of earthquakes;
4. whether the local area is in an earthquake zone and the historical earthquake occurrences; *
5. guidance regarding of how to stock up on emergency food, medicines and supplies; *
6. knowledge of reinforcing houses, home appliances and furniture; *
7. information about earthquake emergency evacuation; *
8. knowledge of self-help, mutual help and emergency care during an earthquake;
9. safe escape routes guidance during an earthquake; *
10. details about nearby safe emergency shelter; *
11. the earthquake emergency plan formulated by the government; *
12. self-psychological guidance knowledge; *
13. none;
14. other___________.

**Q3-1 What types of information do you expect to acquire in emergent period [1] about the Ms. 5.8 earthquake disaster?**
**Q3-2 What types of information do you actually acquire in emergent period about the Ms. 5.8 earthquake disaster?**

1. information regarding the epicenter, magnitude, spread range;
2. information about the cause of the earthquake; *
3. information on the extent of earthquake damage; *
4. personnel casualties; *
5. whether it endangers the safety of the life and property of oneself or relatives and friends; *
6. whether there is a greater danger; *
7. what the government is doing;
8. what I should do;
9. whether anyone is coming to save me;
10. safe evacuation routes; *
11. shelters nearby; *
12. none;
13. other________.

[1] The emergent period refers to the first time after the occurrence of an earthquake, i.e., the moment or day of the earthquake, in a state of emergency, which is the period from the beginning of the dissemination of earthquake information to the beginning of the rapid dissemination of earthquake information.

**Q4-1 What types of information do you expect to acquire in earthquake relief period [2] about the Ms. 5.8 earthquake disaster?**

**Q4-2 What types of information do you actually acquire in earthquake relief period about the Ms. 5.8 earthquake disaster?**

1. information on aftershocks;
2. personnel rescue; *
3. personnel casualties; *
4. resettlement of victims;
5. restoration of transportation and communication;
6. distribution of relief supplies;
7. social relief and donations;
8. information of property losses; *
9. information of secondary disasters; *
10. information of infectious diseases; *
11. reports on volunteers;
12. reports on medical assistance; *
13. reports on disaster relief troops;
14. none;
15. other_________.

[2] The earthquake relief period emphasizes the stage after the public learns the basic information about the earthquake, including both the post-earthquake emergency period and the relief period after the emergency period. That is, the stage when earthquake information spreads rapidly until it subsides.

**Q5-1 What types of information do you expect to acquire in post-disaster reconstruction period [3] about the Ms. 5.8 earthquake disaster?**

**Q5-2 What types of information do you actually acquire in post-disaster reconstruction period about the Ms. 5.8 earthquake disaster?**

1. post-disaster reconstruction plan;
2. Implementation of social donations and relief supplies;
3. living conditions of the affected people; *
4. compensation for disaster losses; *
5. learning and living conditions of children in the disaster area;
6. resettlement of orphans in the disaster area;
7. economic recovery in the disaster area;
8. none;
9. other__________;
10. no concern.

[3] The post-disaster reconstruction period is including both the later period of earthquake relief and the reconstruction period after the earthquake relief. At this time, the disaster is properly resolved, people's lives return to normal, material production is restored, social panic is quelled, and the whole society returns to the state before the disaster.

**Q6 What channels did you use to acquire information in the following four periods?**

| in daily life | in emergent period | in earthquake relief period | in post-disaster reconstruction period |
|---|---|---|---|
| 1. TV reports; | 2. radio broadcasts; | 3. newspapers and magazines; | 4. the internet; |
| 5. mobile phone text messages; | 6. interpersonal communication; | 7. none; | 8. Other |

**Part III. Survey on the socioeconomic and housing characteristics.**

**Q9 What is your gender, please? 1□Male 2□Female**

**Q10 What is your age, please ( )?**

**Q11 What is your educational level, please?**

1□ elementary school education or lower
2□ junior high school
3□ senior high school or special school (including professional high school)
4□ junior college
5□ undergraduate
6□ master degree or higher

**Q12 What is your occupation, please?**

1□ workers / business service workers
2□ enterprise leaders or managers
3□ cadres of public institutions
4□ farmers or migrant workers
5□ general Staff/Clerk/Secretary
6□ public security organs/Military
7□ private or Individual Workers
8□ student
9□ professional technicians/ teachers/doctors
10□ retired personnel
11□ other

| **Q13 What is your family's annual income (after tax), please?** | | |
|---|---|---|
| 1□ CNY 0–20,000 | 2□ CNY 20,000–50,000 | 3□CNY 50,000–100,000 |
| 4□ CNY 100,000–150,000 | 5□ CNY 150,000–200,000 | 6□ Over CNY 200,000 |

**Q14 What is the type of your house? 1□ Purchased by myself; 2□ Rent; 3□ Other**

**Q15 What is the structure of your house?**

| | |
|---|---|
| 1□ Wood or simple structures | 2□masonry-concrete structures |
| 3□ reinforced-concrete structures | 4□ Other |

| **Q16 What is the type of your house?** | | |
|---|---|---|
| 1□ bungalows | 2□ two-storey buildings | 3□ villa |
| 4□ multi-storey buildings | 5□ high-rise buildings | |
